# Mathematical assessment of the effect of traditional beliefs and customs on the transmission dynamics of the 2014 Ebola outbreaks

**DOI:** 10.1186/s12916-015-0318-3

**Published:** 2015-04-23

**Authors:** Folashade B Agusto, Miranda I Teboh-Ewungkem, Abba B Gumel

**Affiliations:** Department of Mathematics and Statistics, Austin Peay State University, Clarksville, 37044 TN USA; Department of Mathematics, Lehigh University, Bethlehem, 18015 PA USA; Simon A. Levin Mathematical, Computational and Modeling Sciences Center, Arizona State University, Tempe, 85287-1904 AZ USA; School of Mathematical and Natural Sciences, Arizona State University, Phoenix, 85069-7100 AZ USA

**Keywords:** Ebola, Community, Hospital, Health-care workers, Quarantine

## Abstract

**Background:**

Ebola is one of the most virulent human viral diseases, with a case fatality ratio between 25% to 90%. The 2014 West African outbreaks are the largest and worst in history. There is no specific treatment or effective/safe vaccine against the disease. Hence, control efforts are restricted to basic public health preventive (non-pharmaceutical) measures. Such efforts are undermined by traditional/cultural belief systems and customs, characterized by general mistrust and skepticism against government efforts to combat the disease. This study assesses the roles of traditional customs and public healthcare systems on the disease spread.

**Methods:**

A mathematical model is designed and used to assess population-level impact of basic non-pharmaceutical control measures on the 2014 Ebola outbreaks. The model incorporates the effects of traditional belief systems and customs, along with disease transmission within health-care settings and by Ebola-deceased individuals. A sensitivity analysis is performed to determine model parameters that most affect disease transmission. The model is parameterized using data from Guinea, one of the three Ebola-stricken countries. Numerical simulations are performed and the parameters that drive disease transmission, with or without basic public health control measures, determined. Three effectiveness levels of such basic measures are considered.

**Results:**

The distribution of the basic reproduction number ($\mathcal {R}_{0}$) for Guinea (in the absence of basic control measures) is such that $\mathcal {R}_{0}\in \;[0.77,1.35]$, for the case when the belief systems do not result in more unreported Ebola cases. When such systems inhibit control efforts, the distribution increases to $\mathcal {R}_{0}\in \;[1.15,2.05]$. The total Ebola cases are contributed by Ebola-deceased individuals (22%), symptomatic individuals in the early (33%) and latter (45%) infection stages. A significant reduction of new Ebola cases can be achieved by increasing health-care workers’ daily shifts from 8 to 24 hours, limiting hospital visitation to 1 hour and educating the populace to abandon detrimental traditional/cultural belief systems.

**Conclusions:**

The 2014 outbreaks are controllable using a moderately-effective basic public health intervention strategy alone. A much higher (>50*%*) disease burden would have been recorded in the absence of such intervention. **2000 Mathematics Subject Classifications** 92B05, 93A30, 93C15.

## Background

Ebola virus disease (EVD), caused by Ebola virus (EBOV) and formerly known as Ebola hemorrhagic fever, is one of the world’s most virulent diseases. The disease, which spreads in human and other mammalian populations, has a case fatality ratio from 25% to 90% in humans [1,2]. The first known case of EVD dates back to 1976, where two outbreaks occurred (in Sudan and in the Democratic Republic of Congo, formerly Zaire; the later outbreak was identified near the Ebola River, where the disease got its name [2,3]). Since then, other outbreaks have occurred, most notably in parts of Central Africa [3]. However, the largest, and most devastating, outbreak of EVD is the 2014 epidemic in three West African countries (Guinea, Liberia and Sierra Leone). This EVD outbreak (believed to have started in Guinea in March 2014 [2]) is the first to have occurred in West Africa [4].

The disease, which also spread to Nigeria (started by an airline passenger, who arrived from Liberia) and Senegal (started by a student from Guinea, who arrived by land transportation) [2,4], spread to other regions outside Africa. For instance, some Ebola-infected patients were flown to the US, France, Germany, Norway, Spain and the UK [5] for health-care delivery. The US diagnosed its first imported travel-related Ebola case in September 2014 (by a person who had travelled to Dallas, Texas, from Liberia). The imported case, who later died of the disease on 8 October 2014, resulted in the infection of two health-care workers who cared for the deceased patient [6]. One of the cases flown to Spain also led to an infection of health-care workers [5]. Additionally, a separate Ebola outbreak, unrelated to the West African outbreaks, occurred in the Democratic Republic of Congo [2]. By 15 October 2014, the case count for the 2014 EVD was 8,997 with 4,493 fatalities [6] (a case fatality ratio of about 50%). It should be mentioned that these estimates include the cases for Nigeria, Senegal, Spain and the USA [7]. These numbers increased to 15,935 and 5,689, respectively (36% case fatality ratio) by 23 November 2014. The latest update, dated 21 January 2015, shows a case count of 21,724 and 8,641 fatalities (representing a case fatality ratio of 40%) [7].

The natural reservoir and host of the EBOV is considered (albeit not yet proven [4]) to be fruit bats of the Pteropodidae family [2]. It is hypothesized that the virus is introduced into the human population when a human comes into contact with the blood, organ secretions or bodily fluids of an animal infected with the EBOV. The incubation period of EBOV is between 2 and 21 days [2,8,9] (although some studies have estimated the most common incubation period to be 8 to 10 days [10]). During the incubation period, the virus infects body cells, replicates and bursts out of the infected cells, producing EBOV glycoproteins that attach to the inside of blood vessels, rendering the blood vessels to be more permeable. The increased permeability causes the blood vessels to leak out blood [8]. The virus also evades the host’s natural defense system, by infecting immune cells, a channel through which it is transported to other body parts and organs, such as the liver, spleen, kidney and brain [8]. The virus can cause these organs to fail, leading to death of the infected human host.

Ebola-infected humans typically exhibit flu-like symptoms during the initial phase of the infection [8], and can have, or progress to, other symptoms such as fever, severe headache, muscle aches, weakness, vomiting, diarrhea, stomach pains, loss of appetite and, at times, bleeding (which may be visible or internal) [8,10,11]. An infected human is infectious (i.e., capable of transmitting the disease to susceptible individuals) at the onset of symptoms [2,8]. Transmission typically occurs when a susceptible human comes into contact with virus-infected fluids, such as blood, bodily secretions (e.g., feces, saliva, vomit, urine, semen and sweat), organs or bodily fluids of an infected human (dead or alive) [2,10]. Contact with such fluids may be as a result of direct contact between susceptible and infected humans, or due to indirect contact with environments contaminated with the aforementioned fluids [2]. Individuals with high risk of exposure to EBOV are the immediate family members of Ebola-infected humans and health-care workers who treat Ebola-infected patients.

In the absence of a cure (a specific treatment) or effective and safe vaccine against the spread of EBOV in humans, anti-Ebola control efforts are mostly restricted to basic public health preventive measures, disease management and treatment of Ebola-related symptoms. Public health preventive measures include approved health-care techniques practiced by health-care workers when dealing with Ebola-infected patients, or, in areas around Ebola-infected patients, educating the public and raising awareness of the disease, quarantine of suspected cases, isolation of symptomatic cases, rapid laboratory diagnostic tests, minimizing contact with bodily fluids, wearing protective equipment by health-care providers and proper handling of individuals who died of the Ebola virus [2,10]. The disease management component of the control strategy (for infected patients) typically entails the administration of intravenous fluids and balancing electrolytes to hydrate the patient, the maintenance of oxygen levels and blood pressure, and possibly a transfusion with blood from a matching Ebola survivor [4].

Recovery from the disease is possible (but the rate of recovery tends to be lower than that of the Ebola-induced death rate [11]). Note that some experimental drugs (such as ZMapp [12] and TKM Ebola [13]) and vaccines are being developed for use in humans (in fact, ZMapp was reportedly used to treat the two American volunteers who contracted the disease while in missionary service in Liberia, although its safety and efficacy have not yet been tested on humans [14]). However, a person’s best chances of survival, following the acquisition of the infection, is early diagnosis (and prompt and effective disease management). This is challenging, however, since the early symptoms of Ebola are similar to those of some other diseases, such as malaria and typhoid fever (diseases that are endemic in the region ravaged by the 2014 EBOV outbreaks [10]). However, using approved laboratory tests, a definitive diagnosis of EBOV can be made [2]. It is known that Ebola infection confers permanent natural immunity (in individuals who have recovered from the disease) against re-infection [15]. Although a human may be clinically cleared of the virus (i.e., is declared to be recovered), a male may, however, still have the virus in his semen for 2 to 3 months [2,15], and it may be found in the breast milk of breast-feeding mothers [15].

A number of mathematical models and statistical methods have been used in an attempt to understand the transmission dynamics of EVD (see for instance [9,16-20], and some of the references therein). In [9], a compartmental mathematical model was used to estimate the number of secondary cases generated by an index case (the basic reproduction number), in the absence or presence of control measures, for the 1995 Congo and 2000 Uganda Ebola outbreaks. The study further highlighted the importance of basic public health control measures, such as public health education, contact tracing and quarantine of suspected cases, and the role such measures can play in reducing the final size of the epidemics. Most recently, the basic reproduction number for the 2014 Ebola outbreak was estimated in [16,17,19-21]. Althaus [16] estimated $\mathcal {R}_{0}$ for EBOV using incidence data and a susceptible-exposed-infectious-recovered (SEIR) type model. The study emphasized the heightening of control measures in the three countries (especially in Liberia). It should, however, be mentioned that the aforementioned studies did not incorporate the role that disease transmission setting (community or health-care facilities) plays in driving or curbing the spread of the disease.

As evidenced by the current EBOV outbreaks in West Africa, the epidemiological setting, in which interaction and transmission between infected and susceptible individuals occur, plays an important role in the spread of the disease [2,10]. For example, health-care workers (doctors, nurses and other paramedic workers who are at the front lines of disease management and control) mostly acquire EVD infection in the hospital setting (or, in general, health-care facilities), while caring for Ebola-infected patients [2,10,11]. They have a high risk of Ebola-induced mortality with 2,400 reported deaths among this group during this 2014 outbreak [22]. Furthermore, the models in [9,16,17,19,20] did not account for disease spread by Ebola-infected deceased individuals (prior to, or during, their burial or cremation), a feature that is known to play a major role in the current outbreaks [2]. Legrand et al. [18] developed a compartmental model, using data from the 1995 Democratic Republic of Congo and 2000 Uganda Ebola epidemic outbreaks. The model allowed for EBOV transmission by infected humans in both the community and the hospital.

Another important feature that plays a critical role in the 2014 EVD outbreaks is traditional/cultural belief systems and customs. For instance, while some individuals in the three Ebola-stricken nations believe that there is no Ebola [23-25], others claim that it is government propaganda to attract more foreign aid dollars [26], control the population or harvest human organs [27]. Furthermore, some susceptible members of the public (including those at high risk of EBOV infection) refuse to be quarantined because of their belief, or fear, that they might be deliberately infected during quarantine [27,28]. There is also a fear that they will not be able to give a loved one who died of Ebola a proper traditional burial (since Ebola-infected humans who die in hospitals are typically cremated [26,27,29], a practice that is not accepted by those who harbor a belief in traditional burial rituals). Adherence to these traditional/cultural beliefs and customs often leads some family members to hide Ebola-infected loved ones (to evade the health-care system), resulting in the development of shadow zones [28], where paramedics cannot visit, and, invariably, resulting in significant underreporting of EVD cases [21,30] (the Centers for Disease Control estimated a potential underreporting correction factor of 2.5 [21]).

The aforementioned modeling studies did not incorporate the effect of traditional belief systems and customs on the transmission dynamics of EVD in the communities (hence, they may have under estimated EVD burden). The purpose of the current study is to assess the role of such belief systems and customs, and health-care settings, on the transmission dynamics of EVD in a population. To achieve this objective, a new deterministic compartmental model, which incorporates the above and other pertinent epidemiological, demographic and biological aspects of EVD, is formulated. The specific goals are to determine the key factors that drive the disease transmission process and to propose effective and affordable strategies to curtail the spread of the disease. The paper is organized as follows. The model is formulated in the section ‘Formulation of compartmental model’, and the worst-case scenario component of the model (in the absence of intervention) is investigated in section ‘Pre-intervention model’. The full model is studied in section ‘Assessment of basic control measures’, where the population-level impact of various effectiveness levels of a basic anti-Ebola public health control strategy are also assessed. Discussion and recommendations stemming from the study (as well as general ones) are given in the Conclusions.

## Methods

### Formulation of compartmental model

This study is based on using a mathematical model, parameterized using data for the 2014 EBOV outbreaks in Guinea, to gain insight into the transmission dynamics of the disease within that nation. Since the 2014 Ebola outbreaks have been ongoing for nearly a year, the model to be designed in this study incorporates demographic effects (and the relevant parameters, *Π*_*C*_ and *μ*_*H*_, as described in Table 1, are estimated using census data from Guinea [31]). The model is formulated by splitting the total population (of Guinea) into two main sub-groups, namely a sub-group of individuals in the community and another for those in health-care settings.

**Table 1:** **Description of the state variables of the model in Figure** **1**

**Variable**	**Description**
*S*_*C*_(*t*)/*S*_*V*_(*t*)	Population of susceptible/visiting-susceptible individuals in the community
*E*_*C*_(*t*)/*E*_*V*_(*t*)	Population of exposed/visiting-exposed individuals in the community
*I*_*CE*_(*t*)/*I*_*CEV*_(*t*)	Population of symptomatic/visiting-symptomatic individuals in the early stage of EBOV infection in the community
*I*_*CN*_(*t*)	Population of non-hospitalized symptomatic individuals
*I*_*CH*_(*t*)	Population of hospitalized symptomatic individuals
*R*_*C*_(*t*),*R*_*CH*_(*t*), *R*_*H*_(*t*),*R*_*RH*_(*t*)	Population of recovered individuals in the community/ health-care workers in the community and hospital
*S*_*H*_(*t*),*S*_*RH*_(*t*)	Population of susceptible/returning-susceptible health-care workers
*E*_*H*_(*t*),*E*_*RH*_(*t*)	Population of exposed/returning-exposed health-care workers
*I*_*H*_(*t*),*I*_*RH*_(*t*)	Population of symptomatic/returning-symptomatic health-care workers
*D*_*C*_(*t*),*D*_*H*_(*t*)	Population of Ebola-deceased individuals in the community and hospital
*C*_*D*_(*t*)	Population of cremated/buried Ebola-deceased individuals

**Figure 1:**
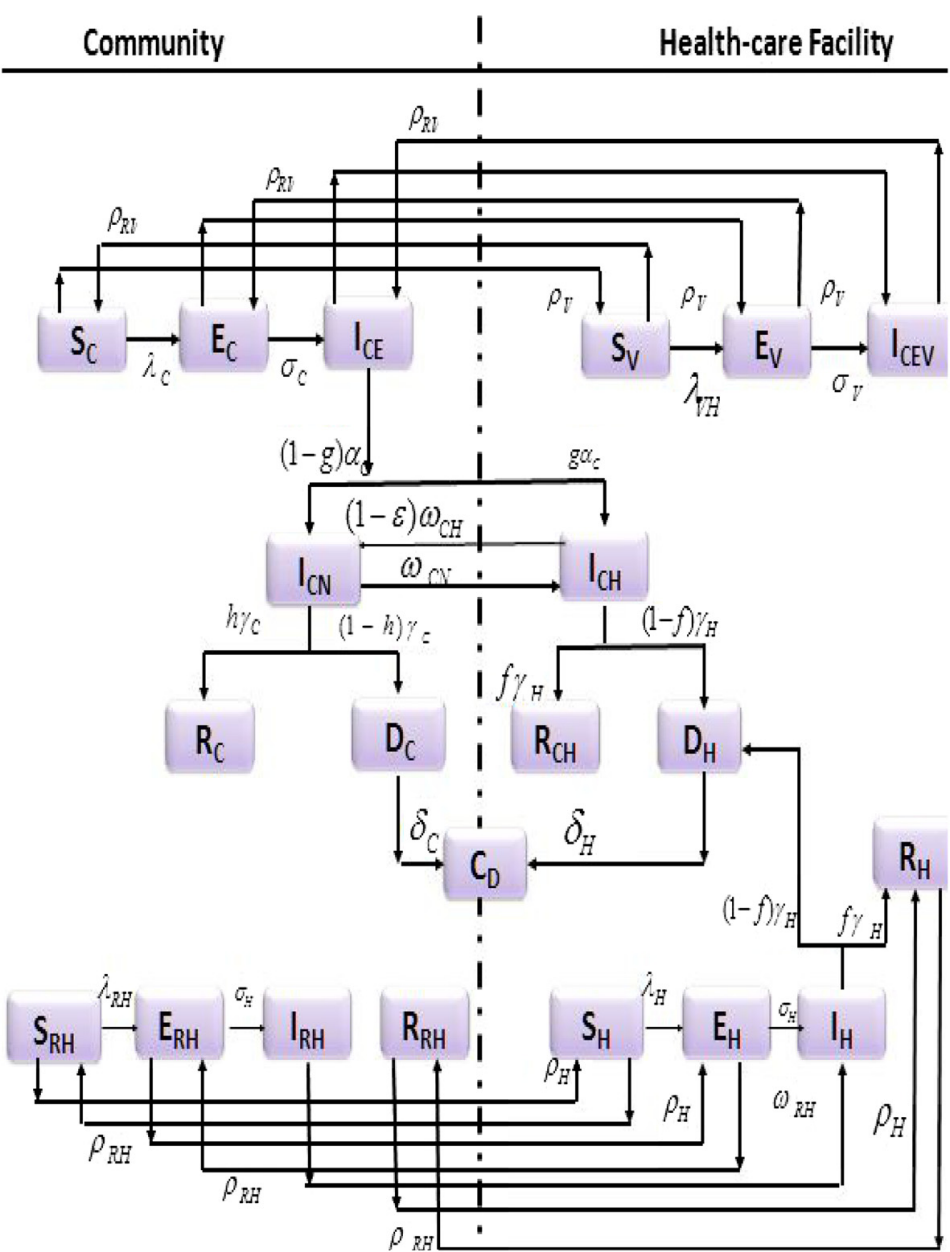
Flow diagram of the model.

The population of individuals in the community consists of individuals visiting loved ones (who are infected with Ebola) in health-care facilities (notably hospitals) and the rest of the general public. This population is further sub-divided into sub-populations of susceptible/visiting-susceptible (*S*_*C*_(*t*)/*S*_*V*_(*t*)), exposed/visiting-exposed (*E*_*C*_(*t*) / *E*_*V*_(*t*)), symptomatic/visiting-symptomatic individuals in the early stage of EVD infection (*I*_*CE*_(*t*)/*I*_*VCE*_(*t*)), non-hospitalized symptomatic (*I*_*CN*_(*t*)), hospitalized symptomatic (*I*_*CH*_(*t*)) and recovered individuals (*R*_*C*_(*t*), *R*_*CH*_(*t*)). The population of individuals in health-care facilities consists of health-care workers in these facilities (as well as those who return to the community at the end of their shift at the hospital). In other words, the population of individuals in the health-care facilities is sub-divided into susceptible/returning-susceptible health-care workers (*S*_*H*_(*t*), *S*_*RH*_(*t*)), exposed/returning-exposed health-care workers (*E*_*H*_(*t*), *E*_*RH*_(*t*)), symptomatic/returning-symptomatic health-care workers (*I*_*H*_(*t*), *I*_*RH*_(*t*)) and recovered/returning-recovered health-care workers (*R*_*H*_(*t*), *R*_*RH*_(*t*)). The model also tracks the dynamics of the Ebola-infected deceased individuals in the community and hospitals (*D*_*C*_(*t*), *D*_*H*_(*t*)) and the cremated/buried Ebola-deceased individuals (*C*_*D*_(*t*)). The equations of the mathematical model are given (and described) in the appendix. A flow diagram of the model is depicted in Figure 1, and the associated state variables and parameters are described in Tables 1 and 2.

**Table 2:** **Description of the state parameters of the model in Figure** **1**

**Parameter**	**Description**
*β*_*C*_,*β*_*H*_	Effective contact (transmission) rate in the community/hospital
*Π*_*C*_,*Π*_*RH*_,*Π*_*V*_,*Π*_*H*_	Recruitment rates
*μ*_*H*_	Natural death rate
*τ*_*C*_, *τ*_*Ci*_, *τ*_*Hi*_ (*i*=1,2)	Modification parameters for infectiousness
*ϕ*_*C*_,*ϕ*_*H*_	Strengths of traditional belief systems and customs in community and hospital
*σ*_*C*_,*σ*_*H*_	Progression rates of symptomatic individuals in the community and hospital
*α*_*C*_,*α*_*H*_	Progression rates of early symptomatic individuals in the community and hospital
*g*	Fraction of symptomatic individuals who are hospitalized
*h*	Fraction of symptomatic non-hospitalized individuals who recovered
*f*	Fraction of symptomatic hospitalized individuals who recovered
*ω*_*CN*_,*ω*_*RH*_	Hospitalization rates of symptomatic individuals in the community and health-care workers
*ω*_*CH*_	Rate of escape from hospitalization
*ε*	Efficacy of hospitalization in preventing the escape of Ebola-infected patients
*ε*_*D*_	Efficacy of hospital-sanctioned burial (burial squad efficacy)
*p*_*D*_	Strength of cultural compliance/acceptance of the burial squad
*γ*_*C*_,*γ*_*H*_	Recovery rates of symptomatic individuals in the community and hospital
*δ*_*C*_,*δ*_*H*_	Cremation/burial rates of Ebola-deceased individuals in the community and hospital
*ρ*_*V*_,*ρ*_*RV*_	Transition rates of visitors between the community and the hospital
*ρ*_*H*_,*ρ*_*RH*_	Transition rates of health-care workers between the community and hospital

Model (4), given in the appendix, extends the Ebola transmission models in [9,16] by (*inter alia*):

- The dynamics of health-care workers are included (i.e., the role of the associated health-care setting).
- The interaction between healthy (susceptible) individuals in a community and infected individuals in a hospital, through visits, are accounted for.
- The effect of traditional (cultural) belief systems and customs that aid EVD transmission (such as the handling of corpses during traditional burial practices, etc.) are accounted for. This also entails the mistrust of members of the community for authority, and fear and stereotypes against seeking medical care (for fear of being quarantined, and/or acquiring infection during quarantine).

Furthermore, model (4) extends that in [18] by incorporating epidemiological compartments for, and dynamics of, health-care workers and members of the general public who visit family members and/or acquaintances in hospitals, in addition to also including the role of traditional belief systems and customs on EBOV transmission dynamics. Although model (4) is parameterized using data from Guinea [16], the parametrization is assumed to be robust enough and applicable to the other two Ebola-stricken nations (Liberia and Sierra Leone).

### Pre-intervention model

Model (4) is, first of all, studied for the special case where no public health interventions (i.e., no basic anti-Ebola control measures and/or disease management in the health-care settings) are implemented in the community. In the absence of such interventions, model (4) reduces to the following basic (worst-case scenario) model (where a dot represents differentiation with respect to time):

(1) $$\begin{array}{@{}rcl@{}} \dot{S}_{C}(t) &=& \Pi_{C} - \lambda_{C}(I_{CE},I_{CN},D_{C})S_{C}(t) - \mu_{H} S_{C}(t),  \\ \dot{E_{C}}(t) &=& \lambda_{C}\left(I_{CE},I_{CN},D_{C}\right)S_{C}(t) - (\sigma_{C}+\mu_{H}) E_{C}(t),\\ \dot{I}_{CE}(t) &=& \sigma_{C} E_{C}(t) - (\alpha_{C}+\mu_{H}) I_{CE}(t),\\ \dot{I}_{CN}(t) &=& \alpha_{C}I_{CE}(t) - (\gamma_{C}+\mu_{H}) I_{CN}(t),\\ \dot{R}_{C}(t) &=& h\gamma_{C}I_{CN}(t)-\mu_{H} R_{C}(t),\\ \dot{D}_{C}(t) &=& (1-h)\gamma_{C} I_{CN}(t)- \delta_{C} D_{C}(t),\\ \dot{C}_{D}(t) &=&\delta_{C} D_{C}(t), \end{array} $$

where

$$\begin{array}{@{}rcl@{}} \lambda_{C}\left(I_{CE},I_{CN},D_{C}\right) & = &\frac{\beta_{C}\phi_{C}\left(I_{CE}+I_{CN}+\tau_{C}D_{C}\right)}{S_{C}+E_{C}+I_{CE}+I_{CN}+R_{C}+D_{C}},\\ \end{array} $$

is the infection rate of the disease (in the community), and all other parameters in *λ*_*C*_ are as defined in Tables 1 and 2. In particular, *β*_*C*_ is the effective contact (transmission) rate, *τ*_*C*_ is a modification parameter that accounts for the assumed reduced infectiousness of Ebola-infected deceased individuals (in comparison to living individuals with Ebola symptoms), and *ϕ*_*C*_≥1 is a modification parameter that accounts for the strength of the traditional belief systems and customs of the community members (that aid Ebola transmission). As stated above, the parameter *ϕ*_*C*_ models, for instance, the belief by some individuals within the Ebola-stricken nations that there is actually no such thing as Ebola [23-25], that Ebola is merely government propaganda [26] or, simply, the fear of being quarantined [27,28] or allowing their loved ones, who have died of Ebola, to be cremated by public health officials (burial squad) [27,29]. The overall effect of the traditional belief systems and customs parameter, *ϕ*_*C*_, in model (1) (or model (4)), is that it leads to the underreporting of new EBOV cases. It is worth re-emphasizing that earlier EBOV models, such as those in [9,16,18], do not incorporate such effects.

The *associated basic reproduction number* [32-35] of model (1), denoted by ${\mathcal R}_{0}$, is given by

(2) $$\begin{array}{@{}rcl@{}} {\mathcal R}_{0} &=& \frac{\beta_{C}\phi_{C}\sigma_{C}}{k_{1} k_{2}k_{3}\delta_{C}}\left[\delta_{C}\left(\alpha_{C}+k_{3}\right) +\tau_{C}\alpha_{C}\gamma_{C}(1-h)\right],  \end{array} $$

where *k*_1_=*σ*_*C*_+*μ*_*H*_, *k*_2_=*α*_*C*_+*μ*_*H*_, *k*_3_=*γ*_*C*_+*μ*_*H*_ and *k*_4_=*σ*_*H*_+*μ*_*H*_. The epidemiological quantity, ${\mathcal R}_{0}$, measures the average number of Ebola cases generated by a typical Ebola-infected individual (living or dead but not buried) introduced into a completely susceptible human population [32-35]. Thus, EBOV can be effectively controlled in the community if the threshold quantity (${\mathcal R}_{0}$) can be reduced to (and maintained at) a value less than unity (i.e., ${\mathcal R}_{0} < 1)$.

#### Interpretation of $\boldsymbol {{\mathcal R}_{0}}$

The basic reproduction number, given by Equation 2, can be rewritten in the following convenient form:

(3) $$  {\mathcal R}_{0} = \frac{\beta_{C}\phi_{C}\sigma_{C}}{k_{1} k_{2}} + \frac{\beta_{C}\phi_{C}\sigma_{C}\alpha_{C}}{k_{1} k_{2}k_{3}}+ \frac{\beta_{C}\phi_{C}\tau_{C}\sigma_{C}\alpha_{C}\gamma_{C}(1-h)}{k_{1}k_{2}k_{3}\delta_{C} }.   $$

The epidemiological quantity, ${\mathcal R}_{0}$, can be interpreted as follows. The first term in Equation 3 measures the average number of new cases generated by symptomatic individuals in the early stage of EBOV infection (*I*_*CE*_). It is the product of the infection rate of susceptible individuals in the community by members of the *I*_*CE*_ group (${\beta _{C}\phi _{C} S_{C}^{*}}/{N_{P}^{*}}=\beta _{C}\phi _{C}$ since $N_{P}^{*}=S_{C}^{*}$), the probability that an exposed individual in the community survives the *E*_*C*_ class and moves to the *I*_*CE*_ class (*σ*_*C*_/*k*_1_) and the average duration in the *I*_*CE*_ class (1/*k*_2_).

The second term in Equation 3 accounts for the average number of new EBOV infections generated by non-hospitalized symptomatic individuals in the community (*I*_*CN*_). It is the product of the infection rate of susceptible individuals by the non-hospitalized symptomatic individuals (${\beta _{C}\phi _{C} S_{C}^{*}}/{N_{P}^{*}}=\beta _{C}\phi _{C}$), the probability that an exposed individual in the community survives the *E*_*C*_ class and transits to the *I*_*CE*_ class (*σ*_*C*_/*k*_1_), the probability that an individual in the *I*_*CE*_ class survives this class and moves to the *I*_*CN*_ class (*α*_*C*_/*k*_2_) and the average duration in the *I*_*CN*_ class (1/*k*_3_).

Finally, the third term in Equation 3 represents the average number of new infections generated by Ebola-infected deceased individuals in the community. It is the product of the infection rate of susceptible individuals by Ebola-deceased individuals (${\beta _{C}\phi _{C} \tau _{C}S_{C}^{*}}/{N_{P}^{*}}=\beta _{C}\phi _{C}\tau _{C}$), the probability that an exposed individual in the community survives the *E*_*C*_ class (*σ*_*C*_/*k*_1_) and moves to the *I*_*CE*_ class, the probability that an individual in the *I*_*CE*_ class survives this class and transits to the *I*_*CN*_ class (*α*_*C*_/*k*_2_), the probability that an individual in the *I*_*CN*_ class did not survive at the end of their time in this class, but died and moved to the *D*_*C*_ class (*γ*_*C*_(1−*h*)/*k*_3_), and the average duration in the cremated/buried class (1/*δ*_*C*_).

The sum of these three terms gives the *basic reproduction number*, ${\mathcal R}_{0}$. The disease can be effectively controlled if ${\mathcal R}_{0}$ is less than unity, and will persist if it exceeds unity.

**Figure 2:**
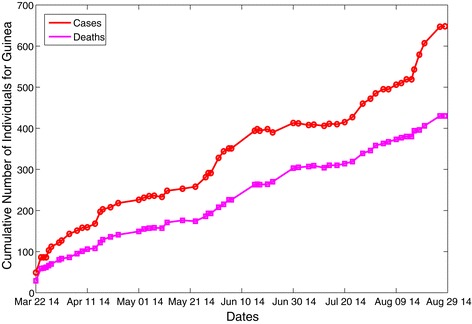
Data fitting of the reported cumulative new cases and EBOV-induced mortality. The fitting used model (1). The data are for the 2014 EBOV outbreaks in Guinea (extracted from the World Health Organization website by Althaus [16]). The parameters fitted are given as *β*_*C*_=0.3045,*σ*_*C*_=0.5239, *α*_*C*_=0.5472, *γ*_*C*_=0.5366 and *ϕ*_*C*_=1.2532. (Approval was given by C. Althaus to use the data cited in [16]).

The numerical value (or range) of the threshold quantity ${\mathcal R}_{0}$ is estimated using the parameter values and ranges tabulated in Table 3. While some of the parameter values in Table 3 were obtained from the literature, others were estimated or fitted based on the EBOV data for Guinea, from 22 March to 29 August 2014 [16] (see Figure 2). For instance, the demographic parameter, *μ*_*H*_, is estimated as *μ*_*H*_=1/58 per year, where 58 years is the average lifespan in Guinea [31]. The other demographic parameter, *Π*_*C*_, is then estimated as follows. Since the total population of Guinea as at 2013 was 11,745,000 [31], we assumed that *Π*_*C*_/*μ*_*H*_, which is the limiting total human population in the absence of the disease, is 11,745,000, so that *Π*_*C*_=202500 per year. Consequently, using these parameter estimates, we show, in this study, that the value of ${\mathcal R}_{0} $ for the 2014 Ebola outbreak in Guinea is ${\mathcal R}_{0} \approx 1.46$. Although this estimate is slightly lower than that reported by Althaus [16] (who used the same data to estimate ${\mathcal R}_{0} \approx 1.51$), it falls within the estimate of ${\mathcal R}_{0} \in \; [1,2]$ given in [16,19,20,36]. The fluctuations in the cumulative data in Figure 2 may be due to the correction of these numbers (by the World Health Organization (WHO)), as more reliable data became available.

**Table 3:** **Values and ranges of the parameters in model (** **4** **) and model (** **1** **)**

**Parameter**	**Baseline value**	**Range**	**Reference**
*β*_*C*_,*β*_*H*_	0.3045	[0.2741 to 0.339]/day	Fitted
*Π*_*C*_	555/day		Estimated using [31]
*Π*_*RH*_,*Π*_*V*_,*Π*_*H*_	400/day	[10 to 800]/day	Variable
*μ*_*H*_	0.00004/day	[ 1/[80×365] to 1/[58×365]]/day	[53,54]
*ψ*_*H*_	(1/10)/day	[1/1,000 to 1]/day	Assumed
*τ*_*C*_, *τ*_*Ci*_, *τ*_*Hi*_, *i*=1,2	0.21/day	[0.1 to 0.5]/day	[55]
*ϕ*_*C*_	1.2532	[1.1282 to 1.3785]	Fitted
*ϕ*_*H*_	1		Fitted
*σ*_*C*_,*σ*_*V*_,*σ*_*H*_	0.5239/day	[0.4715 to 0.5763]/day	Fitted
*α*_*C*_,*α*_*H*_	0.5472/day	[0.4925 to 0.6019]/day	Fitted
*f*,*h*	0.42, 0.48/day	[0.42 to 0.8]/day	[16,18]
*g*	0.5/day	[0.5 to 0.8]/day	[18]
*ω*_*CH*_,*ω*_*CN*_	0.21/day	[0.1 to 0.5], [0.15 to 0.25]/day	Fitted
*ω*_*RH*_	0.5/day	[0.5 to 1.0]/day	Fitted
*ε*	0.21/day	[0.1 to 0.5]/day	Variable
*γ*_*C*_,*γ*_*H*_	0.5366/day	[0.4829 to 0.5903]/day	Fitted
*δ*_*C*_,*δ*_*H*_	(1/2)/day	[1/2 to 1]/day	[18]
*ρ*_*V*_,*ρ*_*RV*_	0.271/hour	[0 to 1/2], 1/7/hour	[55]
*ρ*_*H*_,*ρ*_*RH*_	0.071/hour	[1/16 to 1/12], [1/12 to 1/8]/hour	[55]

#### Sensitivity analysis

**Figure 3:**
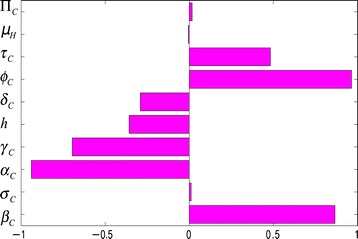
Partial rank correlation coefficient values for model (1). The basic reproduction number ($\mathcal {R}_{0}$) was used as the response function.

Sensitivity analysis [37-39] is carried out, on the parameters of model (1), to determine which of the parameters have the most significant impact on the outcome of the numerical simulations of the model. Figure 3 depicts the partial rank correlation coefficient (PRCC) values for each parameter of the model, using the ranges and baseline values tabulated in Table 3 (with the basic reproduction number, $\mathcal {R}_{0}$, as the response function). It follows from this figure that, in the absence of anti-Ebola public health interventions, the parameters that have the most influence on Ebola transmission dynamics in Guinea are the traditional/cultural/custom belief systems (*ϕ*_*C*_), the progression rate of early symptomatic individuals in the community *α*_*C*_, the effective contact rate (*β*_*C*_) and the recovery rate of symptomatic individuals in the community (*γ*_*C*_). Thus, this study identifies the most important parameters that drive the transmission mechanism of the disease in Guinea. The identification of these key parameters is vital to the formulation of effective control strategies for combatting the spread of the disease. In other words, the results of this sensitivity analysis suggest that a strategy that minimizes the impact of the traditional/cultural beliefs and customs parameter (that is, reduce *ϕ*_*C*_ to a value closer to unity), reduces the progression rate of early symptomatic individuals (decrease *α*_*C*_), reduces the risk of acquisition of Ebola infection in the community (reduce *β*_*C*_) and increases the recovery rate (increase *γ*_*C*_) would be quite effective in curtailing the spread of the disease in the country. Furthermore, these simulations suggest that the 2014 EBOV outbreaks can be effectively controlled using basic (non-pharmaceutical) public health control measures (such as the aforementioned).

Sensitivity analysis was also carried out using the cumulative number of new cases generated by symptomatic individuals in the community at time *t*=360 days (i.e., about a year after the start of the outbreak). In this case, the dominant parameters that positively impact the cumulative number of new cases are the recruitment rate into the community (*Π*_*C*_) and the traditional/cultural/custom beliefs parameter (*ϕ*_*C*_) (these parameters remain dominant even after 18 months). Furthermore, the analysis was implemented using the cumulative number of new cases generated by Ebola-infected deceased individuals, showing, for this case, the dominant parameters to be the modification parameter associated with disease transmission by Ebola-infected deceased individuals (*τ*_*C*_), the fraction of symptomatic individuals who recovered in the community (*h*) and the cremation parameter (*δ*_*C*_); here, too, these parameters remain the dominant ones 18 months after the initial outbreak. Surprisingly, the parameter associated with the detrimental role of the traditional/cultural/custom belief systems (*ϕ*_*C*_) and the recruitment rate (*Π*_*C*_) have only a marginal effect under this scenario. Hence, it follows from the above that the results obtained from the uncertainty/sensitivity analysis are dependent on the response/output function chosen (it is, however, generally accepted that $\mathcal {R}_{0}$ is a very good determinant or predictor of disease burden during an epidemic or disease outbreak).

**Figure 4:**
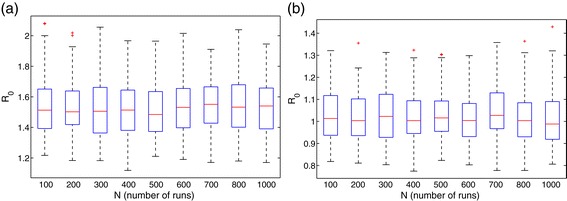
Box plot of ${\mathcal R}_{0}$ for model (1).**(a)** Traditional belief systems and custom parameter, *ϕ*_*C*_=1.5. **(b)** Traditional belief systems and custom parameter, *ϕ*_*C*_=1. Parameter values (baseline) and ranges used are as given in Table 3.

To quantify the expected burden of the disease in the country (under the worst-case scenario), a box plot of the distribution of ${\mathcal R}_{0}$ is generated, using the parameter values and ranges in Table 3 with *ϕ*_*C*_=1.5. The results obtained, depicted in Figure 4a, show the distribution of the reproduction number in the range ${\mathcal R}_{0}\in \, [1.15,2.05]$ (with a mean ${\mathcal R}_{0}\approx 1.6$, suggesting the potential for larger EBOV outbreaks, in comparison to the case where such belief systems and customs had no detrimental effects, where one infected case infects, on average, about 1.6 others). However, when the strength of the traditional beliefs and customs parameter is reduced to *ϕ*_*C*_=1.0 (i.e., people do not harbor detrimental traditional belief systems and customs that aid Ebola transmission), the distribution of ${\mathcal R}_{0}$, depicted in Figure 4b, decreases to ${\mathcal R}_{0}\in \,[0.77,1.35]$, with a mean of ${\mathcal R}_{0}\approx 1$ (corresponding to a much reduced disease burden, in comparison to the former scenario with *ϕ*_*C*_=1.5). It is evident from the box plots in Figure 4 that the disease burden associated with the case where the belief systems and customs are taken into account (Figure 4a) is at least 50% more than that for the case when these systems and customs do not induce any detrimental effect (Figure 4b). Furthermore, it is worth noting that for *ϕ*_*C*_=1.5 (Figure 4a), the box plots are all right-skewed, with the central 50% of the generated ${\mathcal R}_{0}$ values concentrated in the interval [1.36,1.68], with the median close to the mean value, ${\mathcal R}_{0}=1.5$. Thus, large ${\mathcal R}_{0}$ values, such as 2.1 and higher, will likely not be observed. For the case when *ϕ*_*C*_=1.0 (Figure 4b), the box plots are also right-skewed, with the central 50% of the generated ${\mathcal R}_{0}$ values concentrated in the interval [0.9,1.2], with the median around ${\mathcal R}_{0}=1$. Nonetheless, these simulations emphasize the significant role the traditional beliefs and customs parameter (*ϕ*_*C*_) plays in the 2014 EBOV outbreaks in Guinea.

In summary, the aforementioned sensitivity analysis of model (1) suggests that control efforts should be focused on reducing the strength of the traditional beliefs and customs parameter (by reducing *ϕ*_*C*_), increasing recovery rate (by increasing *γ*_*C*_) and reducing transmission (via a reduction in *β*_*C*_). This can be achieved through a variety of ways, such as a public health education/awareness campaign through media and radio advertisements, as well as door-to-door education of members of the community (to desensitize them against harboring such detrimental traditional beliefs and customs). Furthermore, effective measures for curtailing disease transmission by infected people in the community (i.e., minimizing *β*_*C*_) and Ebola-infected deceased individuals (minimizing *τ*_*C*_) must be undertaken. This can be achieved by encouraging the use of protective equipment by health-care workers, proper handling of Ebola-infected deceased individuals (before burial), etc. [29,40,41]. To increase recovery among infected people in the community (i.e., increase the fraction *h*), Ebola clinics and tents should be set up, and the populace encouraged to use them. Since EVD causes high numbers of fatalities, in part due to dehydration of infected individuals [41] and lack of health-care facilities, measures focused on providing adequate resources to such clinics or temporary make-shift tents for visits to patient will help increase the survival chances of Ebola-infected humans. While recruitment (*Π*_*C*_) into the community via immigration (movement) cannot be prevented (except in extreme cases [42]), the public health agencies need to ensure that Ebola test units and clinics are in place at major points of entry, such as airports and border crossings, and to discourage intra-city movement of Ebola-infected individuals [40,43].

#### Role of infectious living humans and Ebola-deceased individuals

In this section, the contributions of EBOV-infected (symptomatic) individuals in the early (*I*_*CE*_) and late (*I*_*CN*_) stages of infectiousness on EBOV burden in the country will be quantified (for the case where no interventions are implemented).

**Figure 5:**
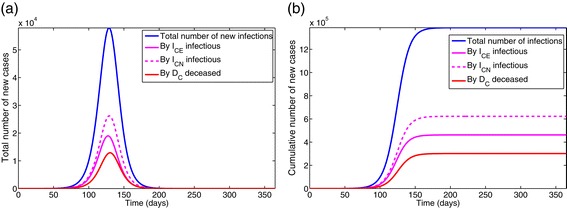
Simulations of model (1).**(a)** Total number of new cases generated by symptomatic living and deceased Ebola-infected individuals. **(b)** Cumulative number of new cases generated by symptomatic living and deceased Ebola-infected individuals. Parameter values used are as given in Table 3.

Figure 5a shows that while the Ebola-infected deceased individuals contribute about 22% of the total number of new infections, individuals in the early infection stage contribute about 33% and those in the late infection stage contribute the bulk of the infections (about 45%). This figure underlines the significance of the role of poor handling of Ebola-infected deceased individuals on the transmission dynamics of the disease in the community.

**Figure 6:**
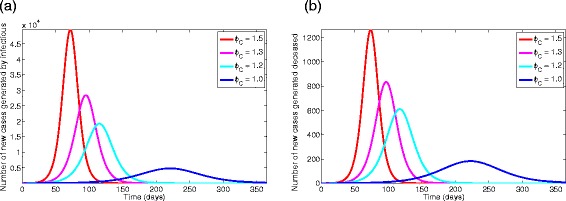
Simulations of model (1) with different values of *ϕ*_*C*_=1.5, 1.3, 1.2, 1.0.**(a)** Total number of new cases generated by symptomatic living individuals. **(b)** Total number of new cases generated by Ebola-infected deceased individuals. Parameter values used are as given in Table 3.

The effect of the traditional belief systems and customs parameter (*ϕ*_*C*_) is further assessed by simulating model (1) using the parameters in Table 3 and various values of *ϕ*_*C*_. The results obtained, depicted in Figure 6, show, as expected, that the total number of new cases generated by symptomatic individuals (and associated peak) increases with increasing values of *ϕ*_*C*_ (Figure 6a). Under this (worst-case) scenario, and with *ϕ*_*C*_=1.5 [21], the number of new Ebola cases peaks at about 49,560 after 72 days of the initial outbreak. Similar results were obtained for the total number of new cases generated by the Ebola-infected deceased individuals (Figure 6b). These simulations further suggest, as expected, that a larger Ebola burden would have been recorded if effective anti-Ebola public health strategies were not implemented (in a timely manner).

In summary, the simulations of the worst-case scenario model (1) show that (in the absence of intervention):

- Traditional belief systems and customs play a vital role in the 2014 Ebola outbreaks in Guinea (this would have resulted in about a 50% increase in the disease burden recorded in Guinea, in the absence of basic public health control measures).
- Ebola-infected deceased individuals contribute about 22% of the total number of new infections, while individuals in the early and later (symptomatic) stages contribute about 33% and 45%, respectively.
- The total number of new cases generated by symptomatic living and Ebola-infected deceased individuals increases with increasing values of the traditional beliefs and customs parameter (*ϕ*_*C*_).
- The 2014 EVD is controllable using (affordable) basic public health control measures that focus on minimizing the strength of the detrimental traditional belief systems and customs in the affected country, increasing the recovery rate and decreasing disease transmission.

### Assessment of basic control measures

**Figure 7:**
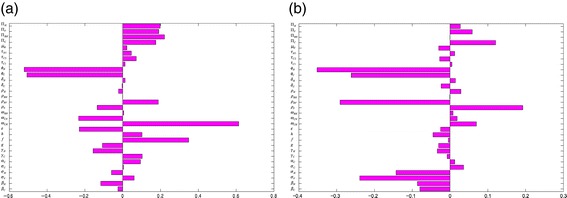
Partial rank correlation coefficient values of model (1). There were two response functions. **(a)** Total number of surviving individuals. **(b)** Total number of symptomatic individuals. Parameter values (baseline) and ranges used are as given in Table 3.

**Figure 8:**
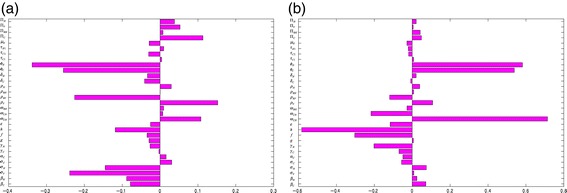
Partial rank correlation coefficient values of model (1). There were two response functions. **(a)** Total number of Ebola-infected deceased individuals. **(b)** Total number of Ebola-infected cremated/buried individuals. Parameter values (baseline) and ranges used are as given in Table 3.

The above analyses were implemented for the worst-case scenario. In practice, however, public health intervention strategies were implemented in an effort to combat effectively the spread of EBOV in the affected countries (and, by extension, globally). In this section, the population-level impact of basic public health intervention strategies, on the disease dynamics in Guinea, is assessed. A sensitivity analysis is, first of all, carried out on the full model (4), with the total number of surviving individuals (susceptible and recovered) as the response (outcome) function. Figure 7a shows the PRCC values for each parameter used in the sensitivity analysis. From this figure, it follows that for model (4), the dominant parameters are the hospital escape rate of symptomatic individuals (*ω*_*CH*_), the parameters for the strength of traditional/cultural/custom beliefs in the community and in health-care settings (*ϕ*_*C*_ and *ϕ*_*H*_), the fraction of symptomatic individuals who recovered in the community (*h*), the efficacy of hospitalization (*ε*) and the hospitalization rate of the non-hospitalized symptomatic individuals (*ω*_*CN*_). A similar analysis was carried out using the total number of symptomatic individuals as the response function, and the dominant parameters in this case (see Figure 7b) are the traditional/cultural/custom beliefs modification parameters for the community and the health-care workers (*ϕ*_*H*_ and *ϕ*_*C*_), the visitors’ mobility rates from the hospital back to the community (*ρ*_*RV*_) and the progression rate of symptomatic individuals in the community and hospital (*σ*_*C*_ and *σ*_*V*_). When the number of Ebola-infected deceased individuals is used as the response function, the dominant parameters are *ϕ*_*H*_, *ϕ*_*C*_, *ρ*_*RV*_, *σ*_*C*_ and *σ*_*V*_ (Figure 8a).

Finally, when the number of Ebola-infected cremated/buried individuals is chosen as the response function, the key parameters (Figure 8b) are the escape rate from hospitalization of symptomatic individuals (*ω*_*CH*_), the fraction of symptomatic individuals who recovered in hospital (*f*), the fraction of symptomatic individuals who recovered in the community (*h*) and the traditional/cultural/custom beliefs modification parameters of the community and the health-care workers (*ϕ*_*C*_ and *ϕ*_*H*_). Again, these results further emphasize the sensitivity of the simulation (sensitivity analysis) results on the response function chosen. These results show that a basic public health strategy that, in addition to the three aspects identified under the worst-case scenario (i.e., decrease *ϕ*_*C*_ to a value less than unity, increase recovery rate (*γ*_*C*_) and decrease transmission rate (*β*_*C*_)), also ensures that hospitalized people do not harbor detrimental traditional beliefs (decrease *ϕ*_*H*_ to a value close to, or equal to, unity), will minimize the hospital escape rate (decrease *ω*_*CH*_ and *ε*), reduce the number and duration of visits in hospitals etc., which will be very effective in curtailing the spread of EBOV.

#### Effectiveness levels of basic intervention strategy

The primary aim of this study is to assess the role of basic (non-pharmaceutical) public health control measures for effective containment of the 2014 EBOV outbreaks. As noted by the WHO on the situation in Liberia, ‘the conventional control interventions are not having adequate impact (in curtailing the spread of EVD) in the country, although they are effective in countries such as Nigeria, Senegal, and the Democratic Republic of Congo with limited transmission’ [44]. Following the results of the sensitivity analysis in section ‘Assessment of basic control measures’ above, the following effectiveness levels of the basic public health control strategy against Ebola are formulated.

#### Low-effectiveness level of the basic public health control strategy

The low-effectiveness level of the anti-Ebola control strategy assumes the strength of the community’s traditional/cultural/custom belief systems to be 1.5 (i.e., *ϕ*_*C*_=1.5). Recall that the beliefs parameter, *ϕ*_*C*_, captures, *inter alia*, the community sentiments and reactions towards the disease, the presence of shadow zones [28] and underreporting [21,30]. It is assumed that hospitalized Ebola-infected individuals do not hold such traditional/cultural beliefs (so that *ϕ*_*H*_=1). Furthermore, due to the relative high value of the beliefs parameter (*ϕ*_*C*_), it is plausible to assume, under this low-effectiveness level of the control strategy, that some symptomatic individuals may choose to escape from the isolation units after a day of hospitalization (i.e., 1/*ω*_*CH*_=1). Additionally, due to the social nature of and strong family ties in the affected communities, it is assumed, for this effectiveness level, that community members visiting their infected loved ones and/or acquaintances in health-care facilities stay at the facilities for an average period of 10 hours (i.e., 1/*ρ*_*V*_=1/*ρ*_*RV*_=10 hours). Health-care workers and returning health-care workers work daily 8-hour shifts (i.e., 1/*ρ*_*H*_=1/*ρ*_*RH*_=8 hours). For this effectiveness level, it is assumed that no transmission-reduction measures are implemented by the health-care workers or visitors in hospitals, and it is further assumed that Ebola-infected deceased individuals transmit at the same rate as living symptomatic individuals (i.e., no extra care in handling Ebola-infected corpses). These assumptions lead to setting 1/*ψ*_*H*_=1/*τ*_*C*1_=1/*τ*_*C*2_=1/*τ*_*H*1_=1.

#### Moderate-effectiveness level of the basic public health control strategy

For the moderate-effectiveness level of the anti-Ebola control strategy, the community’s traditional/cultural/custom beliefs parameter is reduced to 1.2 (i.e., *ϕ*_*C*_=1.2). Here, the hospital (or health-care facility) escape rate of symptomatic individuals is increased to 3 days (i.e., 1/*ω*_*CH*_=3 days). Visiting periods were reduced to 3 hours daily (i.e., 1/*ρ*_*V*_=1/*ρ*_*RV*_=3 hours), and health-care workers and returning health-care workers work daily 16-hour shifts (i.e., 1/*ρ*_*H*_=1/*ρ*_*RH*_=16 hours). For this effectiveness level, the modification parameters for the infectiousness of symptomatic individuals are reduced: 1/*ψ*_*H*_=10 and 1/*τ*_*C*1_=1/*τ*_*C*2_=1/*τ*_*H*1_=100. Lastly, the cremation/burial rates of Ebola-infected deceased individuals (*δ*_*C*_ and *δ*_*H*_) are increased by 50% (i.e., *δ*_*C*_=*δ*_*H*_=0.5×1.5).

#### High-effectiveness level of the basic public health control strategy

For the high-effectiveness level of the anti-Ebola control strategy, the community’s traditional/cultural/custom beliefs parameter is reduced to unity (i.e., these beliefs have no detrimental effect on Ebola transmission dynamics). The escape rate of symptomatic individuals from isolation units is set to 20 days (i.e., 1/*ω*_*CH*_=20 days). The visiting period is reduced to 1 hour daily (i.e., 1/*ρ*_*V*_=1/*ρ*_*RV*_=1 hour), and health-care workers and returning health-care workers work 24-hour shifts (i.e., *ρ*_*H*_=*ρ*_*RH*_=1 day). (It should be stated that requiring health-care workers to work 24-hour shifts may not always be realistic, but the scarcity of such workers in some health-care settings may necessitate this.) Furthermore, the modification parameters for the infectiousness of symptomatic individuals are reduced to 1/*ψ*_*H*_=100 and 1/*τ*_*C*1_=1/*τ*_*C*2_=*τ*_*H*1_=1000. The cremation/burial rates (*δ*_*C*_ and *δ*_*H*_) are increased by 90% (so that *δ*_*C*_=*δ*_*H*_=0.5×1.9).

**Figure 9:**
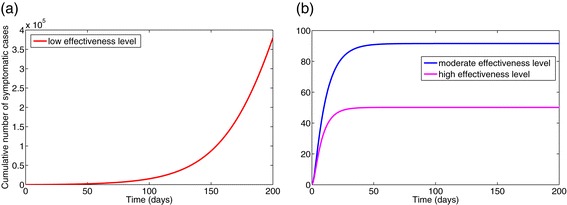
Simulations of model (4). The cumulative number of symptomatic cases generated under various effectiveness levels of the basic public health control strategy is shown. **(a)** Low-effectiveness level. **(b)** Moderate- and high-effectiveness levels. Other parameter values used are as given in Table 3.

**Figure 10:**
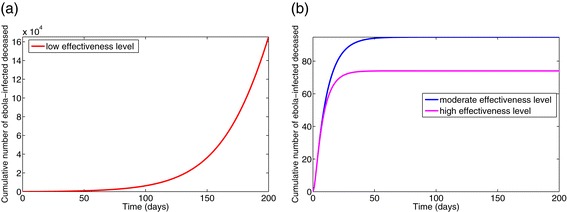
Simulations of model (4). The cumulative Ebola mortality for various effectiveness levels of the basic public health control strategy is shown. **(a)** Low-effectiveness level. **(b)** Moderate- and high-effectiveness levels. Parameter values used are as given in Table 3.
- reducing the duration of visits (of family members and acquaintances) to Ebola isolation units and wards in hospitals/clinics/tents to 1 hour;
- reducing the strength of the community’s detrimental traditional/cultural/custom beliefs, fear, mistrust and anger against public health authorities.

Figure 9a depicts the cumulative number of symptomatic cases generated under the low-effectiveness level of the control strategy over a 200-day period, from which it follows that nearly 380,000 cases would have been recorded. Figure 9b shows a dramatic reduction (to 92 and 50, respectively) under the moderate- and high-effectiveness levels of the control strategy. It is worth noting that although, as expected, the high-effectiveness of the control strategy is far more effective in curtailing Ebola burden in the affected communities, the moderate-effectiveness level of the strategy also resulted in a dramatic decline in the number of cases in comparison to the low-effectiveness level. Similarly, the high-effectiveness level of this strategy is far more effective in reducing the cumulative Ebola-infected mortality (Figures 10). These simulations clearly show that the 2014 Ebola outbreaks are controllable using basic public health control measures, such as the moderate- and high-effectiveness levels of the control strategy described above. In particular, a 90% reduction in Ebola burden can be achieved by implementing basic control measures, such as:

- increasing the duration of health-care workers’ shifts to 24 hours;

It is worth stating that the above simulation results support the success story of Ebola control in Nigeria, a country of over 170 million people and with a more developed public health infrastructure (and largely educated citizenry who are less amenable to harbor such detrimental traditional/cultural belief systems and customs). Nigeria’s first case of Ebola was identified on 20 July 2014 (when a visitor flew into Lagos from Monrovia, Liberia, in search of anti-Ebola medical care) [2,4,45,46]. This resulted in 19 other cases and 8 deaths in total [47]. Nigeria was able to contain the spread of the virus [17,45-48] due, largely, to effective contract tracing of suspected cases, monitoring of traced cases and isolating those with EBOV symptoms. Moreover, the people in the affected area (Lagos State, Nigeria) adhered, strictly, to the anti-Ebola pronouncements and guidelines stipulated by the public health officials (at such a dire time of immense fear) [45]. The WHO declared Nigeria to be Ebola-free on 20 October 2014 [49].

## Results summary and discussion

A new compartmental mathematical model, which stratifies the total population into those in the community and those in health-care facilities, is designed and used to study the 2014 Ebola outbreaks in Guinea. The model incorporates notable crucial features associated with disease transmission, such as the interaction between members of the community and their health-care settings, the role of Ebola-deceased individuals, and traditional belief systems and customs. It is used to assess the population-level impact of basic (non-pharmaceutical) public health control measures (such as proper handling of Ebola-infected patients and Ebola-deceased patients, limiting the duration of family visits to health-care facilities to see infected loved ones etc.).

The study shows that, in the absence of public health interventions, the 2014 EBOV outbreaks would have had a much higher public health burden in Guinea (and, by extension, the other affected countries). The distribution of the basic reproduction number ($\mathcal {R}_{0}$), an epidemiological threshold quantity that measures the spreading capacity of the disease, is estimated to lie in the range [0.77,1.35] when detrimental traditional belief systems and customs are not at play, and within the range [1.15,2.05] if such belief systems and customs are taken into account. Traditional beliefs and customs played a crucial role in fueling the 2014 EBOV outbreaks, since people in the Ebola-stricken communities generally did not adhere to the guidelines of the public health officials. This was because of their mistrust of the authorities, thinking that Ebola was government propaganda or thinking that healthy people who go into quarantine may become deliberately infected while in quarantine. Also, some isolated infected individuals choose to escape isolation because of a fear of being cremated if they die, rather than receiving a proper family burial. Furthermore, it is shown that the incorrect handling of Ebola-deceased individuals contributed to the spread of the disease (estimated to be about 22%; the rest of the infections were generated by symptomatic individuals in the early stage of infection and those who chose not to go to hospital).

This study identifies the main parameters that drove the 2014 EBOV outbreaks during the early (pre-intervention) phase of the disease, namely the traditional/cultural/custom beliefs factor, the transmission rate (effective contact rate) of the disease and the recovery rate of individuals in the community. The identification of these crucial parameters helps in formulating an effective control strategy. For instance, a strategy that minimizes the strength of the detrimental traditional beliefs and customs parameter, as well as reducing the transmission rate and increasing the recovery rate would lead to effective community-wide control of the disease. The strength of the detrimental traditional/cultural/custom belief systems can be reduced via an effective community-wide public health education campaign that involves the local chiefs and community leaders. Transmission can be reduced by taking basic public health measures when caring for Ebola-infected individuals, such as using well-trained health-care professionals and avoiding contact with infected bodily fluids. Transmission can also be reduced by proper handling of Ebola-deceased individuals.

This study shows that the 2014 EBOV outbreaks are controllable using basic (and affordable) public health control measures. In particular, it is shown that a strategy that increases the length of shifts worked by health-care workers caring for Ebola-infected patients to 24 hours, limits the duration of visits of family members and acquaintances to Ebola isolation units and wards to 1 hour and effectively minimizes the strength of the detrimental traditional beliefs and customs (that aid Ebola transmission) could lead to a dramatic reduction (over 90%) of the Ebola burden in the affected communities. We note that while the feasibility of working a 24-hour shift is a tough one to operationalize, it is not unheard of in the health-care profession [50]. Moreover, in situations where there are extreme shortages of health-care professionals during a serious crisis, as in the three countries most affected by the 2014 EVD epidemic, it would not be unusual to see health-care workers working such uncommon shifts [51]. However, it is important to note that requiring a health-care worker to work a 24-hour shift is physically, mentally and emotionally stressful and may result in errors and mistakes in their health-care delivery to patients [51]. In urgent situations and crises were this might occur, plans should be made to ensure that health-care workers who work these shifts only do so for a few days consecutively, and that the nurses and health workers working these shifts organize their schedules and/or patient visits so that they, and their colleagues, get time to rest during the 24-hour period (and further adequate rest at the end of their shift). In other words, this study shows that the 2014 Ebola outbreaks is controllable using basic public health interventions (provided they are of at least a moderate-effectiveness level, and are implemented effectively and consistently).

## Recommendations

We conclude by providing the following list of general recommendations, mostly directly borne out of the simulation results derived from this study, can help in the concerted effort to control effectively the ongoing EBOV outbreaks in West Africa (particularly noting that a cure and an effective and safe vaccine against Ebola transmission in humans remain elusive).

- Public health education and campaign: An effective community-wide public health education campaign, which includes the local leaders (chiefs), has to be embarked upon in an effort to minimize the public mistrust, anger and apprehension against public health authorities and officials who are fighting to end the transmission of EVD. Furthermore, health-care workers must be trained in global best practices, vis-à-vis the proper way to manage, handle and care for Ebola-infected individuals and Ebola-deceased patients (to minimize infection among health-care professionals). This is in line with the finding in this study that detrimental traditional belief systems and customs play a crucial role in the 2014 EBOV outbreaks.
- Creation of Ebola response teams in local communities: Each local community should have an Ebola response team (the grassroots movement team) to help educate the populace about the disease and to identify potential new cases and report them to public health agencies immediately. These local teams must be well trained. Confidence-building measures, to help them build the trust necessary within the communities they serve, must be embarked upon. With a generally weakened health-care system in each of the three Ebola-stricken regions [2], the time it takes to isolate early symptomatic cases may be longer. To limit such a period, and hence minimize underreporting, such a response team can be the ears within the local communities. However, a prompt response from the health-care officials responsible for transporting these potential new cases is necessary for the response team to achieve a meaningful impact. In addition, the response team should serve as a support system to the local members. Such a team should also help convince family members of the need to release their Ebola-deceased relatives to the trained burial teams and help them mourn properly for their loved ones. They can also help minimize factors relating to traditional/cultural beliefs and customs through some of the aforementioned efforts. This will also play an important role in minimizing the detrimental effect of traditional belief systems and customs.
- Preparedness within households: Ebola is one of those rare diseases that forbids the natural love and care, through touch, normally provided to sick loved ones in many cultures. It is difficult for some to see their vulnerable loved ones sick and yet be unable to help. That is generally a hard concept. To avoid such circumstances, each household should have a prepared outline of actions to take if symptoms of EVD become evident within the household. First, health-care officials should be notified. If no one from the health-care system comes to transport the symptomatic individual to a hospital, then while the family members are still able, they should be advised to go to a hospital immediately, avoiding crowds. In the case of children who may not be identified early, one responsible adult, or a parent, should be given a protective suit to transport the child. In the case of late symptomatic individuals, only one designated member in the household should provide support to the sick human, even though the first step should be getting all patients to the hospital or some health-care facility. This will help early detection and hospitalization of cases.
- Social strategy: An unspoken feature that may impact transmission is the social structure associated with the current Ebola outbreak. Families have lost income, schools and businesses have been disrupted and most foreign-owned companies have temporarily closed down, and so the day-to-day functioning and needs of community members have been disrupted. To help cater for the day-to-day needs of communities quarantined, or the potential loss of income from reduced business and cultural activities, aid should be provided to these communities (this would help minimize the strength of the mistrust and fear against public authorities, thereby minimizing EVD cases).
- Global strategy: In each of the three Ebola-stricken countries, health-care workers were overwhelmed [52]. Health-care facilities, which were weak in the first place, are now even more weakened [2]. Thus support, in the form of health-care professionals, from the rest of the world would help to reinforce an overwhelmed health-care system and thus help in the fight against EVD. Given the effects long work hours could have on the efficiency of health-care workers, such global support would help to increase the time period between daily work shifts of health-care workers who are in direct contact with ebola infected patients, there by reducing their chances of becoming infected, which was shown, in this study, to be an effective control tool against EVD.
  Furthermore, support in the form of engineers and construction volunteers is also essential. With a weakened health-care system in the three Ebola-stricken countries [2], the time it takes to isolate early symptomatic cases and move them to a health-care setting may take longer due to a lack of trained professionals to transport the symptomatic humans, or because of a lack of beds at health-care facilities (some of the early symptomatic cases that went to Ebola clinics for consultation were turned down because of a lack of beds [2]). Thus, more health-care tents and units are needed for the isolation and care of symptomatic patients, and support in the form of engineers and construction volunteers would assist in setting up such temporary and permanent health facilities and tents. This would provide space for more symptomatic individuals, reducing their numbers in community settings. Moreover, health-care professionals would be needed to staff these tents.

## Appendix

## Appendix: Formulation of the general Ebola transmission model

We use a compartmental framework to model the transmission dynamics of EVD in a population stratified into two epidemiological settings: those in the community and those within the health-care system. The population of susceptible members of the general public (*S*_*C*_) is generated at the rate *Π*_*c*_ (recruitment or birth). It is further increased by the return of susceptible visitors from the hospital (at a rate *ρ*_*RV*_). The population is decreased by infection (at a rate *λ*_*C*_), natural death (at a rate *μ*_*H*_; this rate is assumed for all epidemiological compartments) and visits to Ebola-infected relatives in health facilities, such as hospitals, clinics, make-shift tent clinics, etc. (at a rate *ρ*_*V*_). The population of exposed (latent infected) members of the community (*E*_*C*_) is generated at the rate *λ*_*C*_ and decreased by development of clinical symptoms of Ebola (at a rate *σ*_*C*_), natural death (at the rate *μ*_*H*_) and visits to infected relatives in health facilities (at the rate *ρ*_*V*_). It is increased by the return of the visitors (at a rate *ρ*_*RV*_).

The population of early infectious individuals (*I*_*CE*_) is generated at the rate *σ*_*C*_ and decreased by progression to the non-hospitalized symptomatic class (at a rate (1−*g*)*α*_*C*_, where *g* is the fraction of these individuals who are hospitalized), hospitalization (at a rate *gα*_*C*_), natural death and visits (at the rate *ρ*_*V*_). It is increased by the return of the visitors (at the rate *ρ*_*RV*_). The population of non-hospitalized symptomatic individuals (*I*_*CN*_) is generated at the rate (1−*g*)*α*_*C*_. It is further increased when hospitalized members of the community escape from hospital (at a rate (1−*ε*)*ω*_*CN*_; where 0<*ε*≤1 is the efficacy of hospitalization to prevent the escape of Ebola-infected patients). This population is decreased by recovery (at a rate *γ*_*C*_), hospitalization (at a rate *ω*_*CN*_) and natural death. The population of recovered members of the community (*R*_*C*_) is generated at a rate *hγ*_*C*_, where *h* is the fraction of non-hospitalized symptomatic individuals who recovered (at the rate *γ*_*C*_; and the remaining fraction, 1−*h*, is deceased). It is reduced by natural death. The population of members of the community who died of Ebola (*D*_*C*_) is generated at the rate (1−*h*)*γ*_*C*_ and is decreased by cremation (at a rate *δ*_*C*_).

The equations for the dynamics of health-care workers (those in hospitals, or health-care facilities in general, and health-care workers who return to the community at the end of their shift) are similarly derived (and not repeated here).

(4) $$\begin{array}{@{}rcl@{}} \dot{S}_{C}(t) &=& \Pi_{C} - \lambda_{C}(I_{CE},I_{CN},I_{RH},D_{C})S_{C}(t) - \mu_{H} S_{C}(t)\\ && - \rho_{V} S_{C} + \rho_{RV}S_{V},  \\ \dot{E_{C}}(t) &=& \lambda_{C}(I_{CE},I_{CN},I_{RH},D_{C})S_{C}(t) - (\sigma_{C}+\mu_{H}) E_{C}(t) \\ &&- \rho_{V} E_{C}(t) + \rho_{RV}E_{V}(t),\\ \dot{I}_{CE}(t) &=& \sigma_{C} E_{C}(t) - (\alpha_{C}+\mu_{H}) I_{CE}(t) - \rho_{V} I_{CE}(t)\\ &&+ \rho_{RV}I_{CEV}(t),\\ \dot{I}_{CN}(t) &=& (1-g)\alpha_{C}I_{CE}(t) + (1-\varepsilon)\omega_{CH} I_{CH}(t)\\ &&-(\gamma_{C}+\omega_{CN}+\mu_{H}) I_{CN}(t),\\ \dot{R}_{C}(t) &=& h\gamma_{C}I_{CN}(t)-\mu_{H} R_{C}(t),\\ \dot{D}_{C}(t) &=& (1-h)\gamma_{C} I_{CN}(t)- \delta_{C} D_{C}(t), \end{array} $$

$$\begin{array}{@{}rcl@{}} \dot{S}_{RH}(t) &=& \Pi_{RH} \,-\, \lambda_{H}(I_{CE},I_{CN},I_{RH},D_{C})S_{RH}(t) \,-\, \mu_{H} S_{RH}(t)\\ &&- \rho_{RH}S_{RH}(t) + \rho_{H}S_{H}(t),\\ \dot{E}_{RH}(t) &=& \lambda_{H}(I_{CE},I_{CN},I_{RH},D_{C})S_{RH}(t) \,-\, (\sigma_{H}\,+\,\mu_{H}) E_{RH}(t)\\ && - \rho_{RH}E_{RH}(t) + \rho_{H}E_{H}(t), \\ \dot{I}_{RH} (t) &=& \sigma_{H} E_{RH}(t) - (\gamma_{H}+\mu_{H}) I_{RH}(t) - \omega_{RH} I_{RH}(t),\\ \dot{R}_{RH}(t) &=& \gamma_{H} I_{RH}(t) - \mu_{H} R_{RH} - \rho_{RH}R_{RH}(t) + \rho_{H}R_{H}(t),\\ \dot{I}_{CH}(t) &=& g\alpha_{C}I_{CE}(t) + \omega_{CN} I_{CN}(t) - [(1-\varepsilon)\omega_{CN}+\gamma_{H}\\ &&+\mu_{H}] I_{CH}(t),\\ \dot{R}_{CH}(t) &=& f\gamma_{H}I_{CH}(t) - \mu_{H} R_{CH}(t),\\ \dot{S_{V}}(t) &=& \Pi_{V} - \lambda_{H}(I_{CEV},I_{CH},I_{H},D_{H}) S_{V}(t) - \mu_{H} S_{V}(t)\\ && - \rho_{RV}S_{V}(t) + \rho_{V} S_{C}(t),\\ \dot{E}_{V}(t) &=& \lambda_{H}(I_{CEV},I_{CH},I_{H},D_{H}) S_{V}(t) - (\sigma_{V}+\mu_{H}) E_{V}(t)\\ && - \rho_{RV}E_{V}(t) + \rho_{V} E_{C}(t),\\ \dot{I}_{CEV}(t) &=& \sigma_{V}E_{V}(t)\! - \!\mu_{H} I_{CEV}(t) \!- \!\rho_{RV}I_{CEV}(t) \,+\, \rho_{V} I_{CE}(t),\\ \dot{S}_{H}(t) &=& \Pi_{H} - \lambda_{H}(I_{CEV},I_{CH},I_{H},D_{H})S_{H}(t) - \mu_{H} S_{H}(t)\\ && - \rho_{H}S_{H}(t) + \rho_{RH}S_{RH}(t),\\ \dot{E}_{H}(t) &=& \lambda_{H}(I_{CE},I_{CH},I_{H},D_{H})S_{H}(t) - (\sigma_{H}+\mu_{H}) E_{H}(t)\\ && - \rho_{H}E_{H}(t) + \rho_{RH}E_{RH}(t),\\ \dot{I}_{H} (t) &=& \sigma_{H} E_{H}(t) + \omega_{RH} I_{RH}(t) - (\gamma_{H}+\mu_{H}) I_{H}(t),\\ \dot{R}_{H}(t) &=& f\gamma_{H} I_{H}(t) - \mu_{H} R_{H}(t) + \rho_{RH}R_{RH}(t) \,-\, \rho_{H}R_{H}(t),\\ \dot{D}_{H}(t) &=&(1-f)\gamma_{H} I_{CH}(t) + (1-f)\gamma_{H} I_{H}(t) - \delta_{H}D_{H}(t),\\ \dot{C}_{D}(t) &=&\delta_{C} D_{C}(t) + \delta_{H}D_{H}(t), \end{array} $$

where,

$$\fontsize{8.8}{12}{\begin{aligned} \lambda_{C}(I_{CE},I_{CN},I_{RH},D_{C})& = \frac{\phi_{C}\beta_{C}(I_{CE}+I_{CN}+\tau_{C1}I_{RH}+\tau_{C2}D_{C})}{N_{P}}, \\ \lambda_{H}(I_{CEV},I_{CH},I_{H},D_{H}) & = \frac{\psi_{H}\phi_{H}\beta_{H}(I_{CEV}+I_{CH}+I_{H}+\tau_{H1}D_{H})}{N_{P}},\\ \end{aligned}} $$

with *N*_*P*_=*S*_*C*_+*E*_*C*_+*I*_*CE*_+*I*_*CN*_+*R*_*C*_+*D*_*C*_+*S*_*RH*_+*E*_*RH*_+*I*_*RH*_+*R*_*RH*_+*I*_*CH*_+*R*_*CH*_+*S*_*V*_+*E*_*V*_+*I*_*CEV*_+*S*_*H*_+*E*_*H*_+*I*_*H*_++*R*_*H*_+*D*_*H*_+*C*_*D*_.

## Abbreviations

EBOV: Ebola virus
EVD: Ebola virus disease
PRCC: partial rank correlation coefficient
WHO: World Health Organization
